# Target-site resistance mutations (*kdr* and *RDL*), but not metabolic resistance, negatively impact male mating competiveness in the malaria vector *Anopheles gambiae*

**DOI:** 10.1038/hdy.2015.33

**Published:** 2015-04-22

**Authors:** N Platt, R M Kwiatkowska, H Irving, A Diabaté, R Dabire, C S Wondji

**Affiliations:** 1 Department of Vector Biology, Liverpool School of Tropical Medicine, Liverpool, UK; 2 IRSS/Centre Muraz, BP 390 Bobo-Dioulasso, Burkina Faso, Africa

## Abstract

The implementation of successful insecticide resistance management strategies for malaria control is currently hampered by poor understanding of the fitness cost of resistance on mosquito populations, including their mating competiveness. To fill this knowledge gap, coupled and uncoupled *Anopheles gambiae s.l.* males (all M form (*Anopheles coluzzii*)) were collected from mating swarms in Burkina Faso. This multiple insecticide resistant population exhibited high 1014F *kdr*^*R*^ allele frequencies (>60%) and *RDL*^*R*^ (>80%) in contrast to the *Ace-1*^*R*^ allele (<6%). *Kdr* heterozygote males were more likely to mate than homozygote resistant (OR=2.36; *P*<0.001), suggesting a negative impact of *kdr* on *An. coluzzii* mating ability. Interestingly, heterozygote males were also more competitive than homozygote susceptible (OR=3.26; *P*=0.006), suggesting a heterozygote advantage effect. Similarly, heterozygote RDL^R^/RDL^S^ were also more likely to mate than homozygote-resistant males (OR=2.58; *P*=0.007). Furthermore, an additive mating disadvantage was detected in male homozygotes for both *kdr/RDL-*resistant alleles. In contrast, no fitness difference was observed for the *Ace-1* mutation. Comparative microarray-based genome-wide transcription analysis revealed that metabolic resistance did not significantly alter the mating competitiveness of male *An. coluzzii* mosquitoes. Indeed, no significant difference of expression levels was observed for the main metabolic resistance genes, suggesting that metabolic resistance has a limited impact on male mating competiveness. In addition, specific gene classes/GO terms associated with mating process were detected including sensory perception and peroxidase activity. The detrimental impact of insecticide resistance on mating competiveness observed here suggests that resistance management strategies such as insecticide rotation could help reverse the resistance, if implemented early.

## Introduction

Resistance to most insecticide classes used in public health is spreading in malaria vectors in Africa (WHO, 2012). There is a fear that such resistance is likely to increase due to ongoing scaling up of vector control interventions such as long-lasting impregnated nets (LLINs) and indoor residual spraying (IRS). Successful management of resistance will require a good understanding not only of the mechanisms of resistance but more importantly its impact on key traits of mosquito biology, ecology and behavior. Many resistance management strategies such as rotation of insecticides are based on the assumption that resistance induces a fitness cost on mosquitoes such that, in the absence of selection pressure from the specific insecticide, the mosquito population will rapidly revert to susceptibility. However, little is currently known on such fitness costs in field populations of malaria vectors.

It is generally acknowledged that mutations responsible for adaptation to a new environment are associated with a fitness cost (Arnaud and Haubruge, 2002; Higginson *et al.*, 2005). Similarly, mutations conferring resistance may divert resources away from fitness enhancing characteristics or cause disruption of normal physiological functions (Rowland, 1991a; McCarroll *et al.*, 2000; Higginson *et al.*, 2005). In the presence of insecticide, the detrimental effects of this reallocation of resources are outweighed by the fitness advantages, but the removal of this selection pressure could place resistant mosquitoes at a competitive disadvantage. One study demonstrated the disadvantage in competitive mating ability of *Culex pipiens* males with the *Ace1*^*R*^ genotype, when compared with susceptible males, highlighting its potential impact on the spread and persistence of resistant alleles (Berticat *et al.*, 2002). Another study, using *An. gambiae* laboratory strains, demonstrated fewer copulations in dieldrin-resistant males when compared with their susceptible counterparts (Rowland, 1991a). The author suspected this had a greater impact on reversion to susceptibility than the lowered fecundity of resistant females, highlighting the importance of such a fitness cost. However, genotyping was based on the progeny phenotype, without identification of the underlying resistance mechanisms. Other resistant insects also demonstrate mating costs, such as the pink bollworm with less first male paternity (Higginson *et al.*, 2005). In contrast, a study regarding malathion resistance in the beetle *Tribolium castaneum* (Arnaud and Haubruge, 2002) suggested that this resistance enhanced male reproductive success. If such fitness advantage was present in resistant malaria vectors, it will represent a serious challenge to any malaria control program as resistance could increase even in the absence of any insecticide application. This will prevent the use of resistance management strategies such as rotation, which is based on the hypothesis that resistance will decrease in the absence of selection pressure. Little information is currently available on the impact of insecticide resistance on the mating ability of natural populations of malaria vectors in Africa. Filling this knowledge gap is essential to improve the design and implementation of suitable resistance management strategies.

Significant progress has been made recently in the understanding of mating behavior in malaria vectors such as *An. gambiae* for which aerial male aggregation has an important role in mosquito mating. *An. gambiae* swarms, composed entirely of males, provide the opportunity for insemination of mate-searching females (Diabate *et al.*, 2006). Mating in *An. gambiae s.s.* is confined to a short period at dusk, with males always swarming before and disbanding after copulation (Charlwood and Jones, 1980). Females approach a swarm, promptly acquire a male and leave *in copula* (Charlwood and Jones, 1980; Diabate *et al.*, 2006). Such mating behavior provides the opportunity to compare mosquitoes that successfully mated to those that could not mate and assess specific traits, such as presence of insecticide resistance alleles.

Burkina Faso, notably the region of Vallée du Kou (VK), has been the focus of several studies on mosquito mating behavior (Diabate *et al.*, 2003; Diabate *et al.*, 2006; Dabire *et al.*, 2013; Sawadogo *et al.*, 2014) and also on insecticide resistance (Dabire *et al.*, 2008; Kwiatkowska *et al.*, 2013). Indeed, extensive resistance to several insecticides has been previously reported in the Vallée du Kou region with several underlying mechanisms including target site resistance (*kdr*, *Ace-1* and *RDL*) and also metabolic resistance with involvement of detoxification enzyme families such as cytochrome P450 genes and glutathione-*s*-transferases (Dabire *et al.*, 2008; Kwiatkowska *et al.*, 2013). The widespread distribution and high frequency of insecticide resistance and the presence of multiple resistance mechanisms render the Vallée du Kou ideal to study the impact of insecticide resistance on mating success.

Therefore, this study was undertaken to determine the impact of insecticide resistance on competitive mating ability in field populations of *An. gambiae s.s.*, with analysis of all known target site and metabolic resistance mechanisms. Genotype distribution of *kdr*, *Ace-1* and *RDL* resistance mutations was compared between coupled and uncoupled males within the mating swarms to determine the impact on mate selection by females, whereas a comparative genome-wide expression profiling was performed to determine the impact of metabolic resistance on mating competiveness.

## Materials and Methods

### Study site

The Vallée du Kou (VK) (40° 25' W, 11° 25' N) in Burkina Faso comprises seven villages and 1200 hectares of agricultural land, surrounded by humid savannah. Permanent irrigation by the Kou River makes the land ideal for rice agriculture and the water source is robust enough to support two harvests annually. Although rice requires few insecticides, surrounding cotton agriculture and the recent introduction of vegetables into rice paddies necessitate intensive pesticide use, with a marked impact on insecticide resistance (Dabire *et al.*, 2008). Mosquito density exhibits huge seasonal variation, with malaria transmission peaking during the rains from June to October, corresponding with up to 200 bites/person/night (Dabire *et al.*, 2008). Both M (now *An. coluzzii*) and S (*An. gambiae s.s*.) molecular forms of *An. gambiae* are present during rains, in contrast to the dry season from November to May, when only *An. coluzzii* is detected. VK3 is a village in the centre of the rice growing area. As one of the smaller villages in the Vallée du Kou, it has ~600 inhabitants, mostly farmers, and animals such as sheep, pigs, goats and a few cows are also present. In addition to rice other crops include maize, bananas and okra. These crops are incorporated into the rice field areas.

### Swarm collections

Swarm collections were undertaken in VK3 on 10 evenings in April 2010. Swarms were located and coded, mosquitoes identified according to the standard keys of Gillies and Coetzee (1987) and global positioning system (GPS) co-ordinates recorded. All mating *An. gambiae s.l.* couples were extracted from the swarms using sweep nets, and each couple manually aspirated into a clean container. As darkness fell and coupling ceased, samples of the remaining males (that most likely did not mate) in the same swarms were taken using sweep net, with a target of thirty uncoupled males from each swarm on each occasion, which were stored according to swarm. Within 1 h of collection, all captured mosquitoes were killed and completely immersed in RNAlater solution and kept at 4 °C for 24 h to allow the mosquitoes to be fully soaked into the solution and then stored at −20 °C. Both couples and uncoupled males were coded according to the swarm and date.

### Indoor female collections and F_1_ rearing

For the purpose of obtaining a control sample not affected by mating competition, virgin males were generated from indoor-resting blood-fed females in VK3, collected using manual aspirators. Collected female mosquitoes were transferred to the insectary and provided with cotton wool soaked in sucrose solution and let to lay eggs. Larvae were reared using fish food (TetraMin) and, on emergence of the adults, males and females were separated and fed with glucose-moistened cotton wool. Three days following emergence virgin males were stored in RNAlater.

### Bioassays

To establish the resistance levels of the *An. gambiae* population in VK3 during this study, WHO adult bioassays were undertaken in 2- to 5-day-old, non-blood-fed female mosquitoes, using filter paper impregnated with 4% DDT, 0.75% permethrin, 0.1% bendiocarb and 4% dieldrin following WHO protocol (WHO, 1998) and mortality rate recorded after a 24 -h recovery period. The Kisumu susceptible strain of *An. gambiae s.s.* was used as a reference susceptible strain, whereas F_1_ females from VK3 were used as controls alongside each bioassay.

### Species and molecular form identification

Genomic DNA was extracted using the LIVAK technique (Livak, 1984) from 50 randomly selected males and females from collected couples, 50 uncoupled males and 50 virgin males. Uncoupled males were randomly selected from those swarms represented by couples, to allow direct comparison. Species ID of these specimens and the molecular form of *An. gambiae s.s.* specimens were identified using the SINE PCR protocol (Santolamazza *et al.*, 2008).

### Genotyping of target site mutations using the pyrosequencing method

Fifty DNA samples from each VK3 mosquito group (coupled males and females, uncoupled males from swarms and virgin F_1_ males) were genotyped for three target site mutations. For the knockdown resistance (*kdr*) mutation, conferring pyrethroid and DDT resistance, both L1014F and L1014S *kdr* mutations were genotyped in a same assay. The same was done for the G119S *Ace-1* mutation, conferring carbamate/organophosphate resistance, and also for the A296S *RDL* mutation conferring dieldrin resistance, as described previously (Wondji *et al.*, 2007; Kwiatkowska *et al.*, 2013). Pyrosequencing reactions were carried out as described previously (Wondji *et al.*, 2007).

A *χ*^2^-test was used to compare the frequency distribution of *kdr*, *Ace-1* and *RDL* genotypes between the coupled males, uncoupled males, coupled females and F_1_ virgin males. In addition, association between genotypes of these mutations and mating success was assessed by estimating the odds ratios between mated and unmated mosquitoes and the statistical significance, using Fisher's exact test.

### Comparative genome-wide transcription profiling of coupled and uncoupled males to assess impact of metabolic resistance

The 8 × 15 K Agilent microarray chip (A-MEXP-2196) (Mitchell *et al.*, 2012) was used to detect genes differentially expressed between the coupled male mosquitoes and the uncoupled to assess the impact of metabolic resistance mechanism on mating competitiveness. Each array contains 60mer probes designed from all 13 000 transcripts of the Ensembl P3.5 *An. gambiae* genome annotation, plus additional probes from detoxification genes (Mitchell *et al.*, 2012).

Total RNA was extracted from three batches of 10 pooled mosquitoes from the coupled and 10 pooled uncoupled males of VK3. The PicoPure RNA isolation kit (Arcturus, Foster City, CA, USA) was used according to the manufacturer's instructions. The quantity and quality of extracted RNA were assessed using a NanoDrop ND1000 spectrophotometer (Thermo Fisher, Waltham, MA, USA) and Bioanalyzer (Agilent, Santa Clara, CA, USA), respectively. Complementary RNA (cRNA) for each sample was amplified using the Agilent Quick Amp labeling Kit (two-color) following the manufacturer's protocol. Labeled cRNA from the coupled and uncoupled males was hybridized to the arrays for 17 h at 65 °C according to the manufacturer's protocol. Five hybridizations between cRNA from coupled and uncoupled samples were carried out.

Microarray data were analyzed using Genespring GX 12.0 software (Agilent). To identify differentially expressed genes, a cutoff of 1.5-fold-change was chosen and a statistical significance of *q*<0.05 for the Storey with bootstrapping correction for multiple testing. Enrichment analysis was carried out using the Blast2Go software (Conesa *et al.*, 2005; Gotz *et al.*, 2008) and the DAVID functional program (Huang da *et al.*, 2009) to detect the major Gene Ontology (GO) terms overrepresented among the set of probes up- or downregulated in the coupled and uncoupled samples in comparison with the entire microarray chip using the false discovery rate (FDR) test for statistical significance.

### qRT-PCR comparative transcription profile of insecticide resistance genes between mated and unmated males

The expression profile of six of the most overexpressed detoxification genes previously associated with metabolic resistance in VK through microarray analysis (Kwiatkowska *et al.*, 2013) was further assessed by qRT-PCR to validate their differential expression profile between mated and unmated mosquitoes (genes names and primer sequences are given in Supplementary Table S1). One microgram of total RNA from each of the three biological replicates for mated and unmated mosquitoes was used as a template for cDNA synthesis using Superscript III (Invitrogen, Loughborough, UK) with oligo-dT20 and RNase H (New England Biolabs, Ipswich, MA, USA), according to the manufacturer's instructions. A serial dilution of cDNA was used to establish standard curves for each gene to assess PCR efficiency and quantitative differences between samples. The qPCR amplification was performed using a MX 3005 real-time PCR system (Agilent) with Brilliant III Ultra-Fast SYBR Green QPCR Master Mix (Agilent) as described previously (Kwiatkowska *et al.*, 2013). The relative expression and fold-change of each target gene in mated relative to unmated was calculated according to the 2^−ΔΔCT^ method incorporating PCR efficiency (Schmittgen and Livak, 2008) after normalization with the housekeeping genes *rsp7*, encoding ribosomal protein S7 (AGAP010592) and elongation factor gene (AGAP005128).

## Results

### Swarm observation and collections

Eight swarm sites were identified in VK3 with GPS co-ordinates recorded for each (Supplementary Table S2) and used for collections. Swarms formed roughly 5 min after sunset with one or two males in a zigzag flight pattern, gradually growing in number. Swarms remained stationary at 1–3 m above markers, including a pigsty, a rubbish heap and a well, and were observed at the same sites each evening. Mosquito density varied enormously, demonstrated by swarm size and coupling frequency, with 1–43 (median=7) couples captured per collection. One hundred and thirteen couples were sampled from these locations during 10 collections in April 2010. A sudden increase in density and coupling occurred 9 days after the opening of irrigation systems, provided more breeding sites for the mosquitoes. Uncoupled (single) males were sampled in each swarm with 6–73 (median=28) uncoupled males collected per swarm. Only two uncoupled females were captured during the entire study. Collection data are shown in Supplementary Table S3.

The SINE PCR carried out on all of the 50 mosquitoes in each of the four sample groups indicated that they were all *An. gambiae s.s.* from the M molecular form, now re-classified as *An. coluzzii*.

### Resistance pattern of *An. coluzzii* in VK3

Although resistance in the Vallée du Kou is well documented, bioassays were performed to establish the resistance levels in the VK3 *An. coluzzii* population during this study. In total, 290 *An. gambiae* females laid viable eggs that successfully generated F_1_ adults for the bioassays. The VK3 population was highly resistant to both permethrin and DDT with only 12 and 6% mortality after 1 h exposure, respectively. Moderate resistance was observed against the carbamate bendiocarb, with 91% mortality (Supplementary Table S4).

### Impact of target site resistance on mating competiveness

#### Impact of *L1014 F kdr* genotypes on mating success

**Figure 1:**
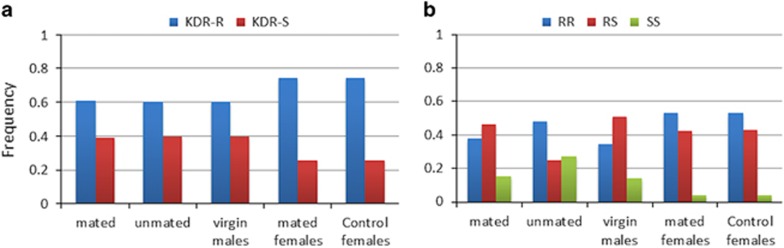
Impact of L1014F *kdr* mutation on male mating success. Distribution of *kdr* alleles (**a**) and genotypes (**b**) between coupled and uncoupled males in comparison with control mosquitoes.

High frequencies of the 1014F *kdr*^*R*^ resistant allele were detected in all samples, ranging from 60.2% in virgin males to 74.5 in mated females (Table 1; Figure 1a). Direct comparison of the genotype distribution using a ared test revealed statistically significant (*P*<0.001) differences between all groups of mosquitoes, except between coupled and virgin males (Supplementary Table S5; Figure 1b). An assessment of the association of each genotype with mating success was carried out by estimating the relative odds ratio (OR) between coupled and uncoupled males. The heterozygote genotype (RS) showed a significantly higher chance of being mated than resistant homozygote mosquitoes (RR) (OR=2.36; *P*<0.001) as higher frequency of heterozygote mosquitoes was observed in coupled males (46.7%) than in uncoupled (25%) (Table 2). In contrast, a significantly higher frequency of resistant homozygotes was detected in the uncoupled mosquitoes (47.9%) than in mated ones (37.8%). Surprisingly, the heterozygote genotype was significantly more associated with mating success than the homozygote-susceptible genotype (OR=3.26; *P*=0.006), suggesting that heterozygote mosquitoes may have a fitness advantage over wild mosquitoes. No significant difference was observed between homozygote-resistant RR and homozygote-susceptible SS mosquitoes.

#### Impact of the G119S Ace-1 genotypes on mating success

In line with the low bendiocarb resistance observed in VK3, only a very low frequency of the resistant *Ace-1*^*R*^ allele was detected, ranging from 1.6% in virgin males to 5.9% in coupled males (Supplementary Figure S1A). No homozygous resistant mosquitoes were detected and only a low frequency of heterozygote genotypes observed (Supplementary Figure S1B). No significant difference was noted in the distribution of genotypes between coupled and uncoupled males (*χ*^2^=1.18, *P*>0.05) and no association was observed with mating success between RS and SS genotypes (OR=0.72; *P*>0.05).

#### Impact of *A296S RDL* genotypes on mating success

**Figure 2:**
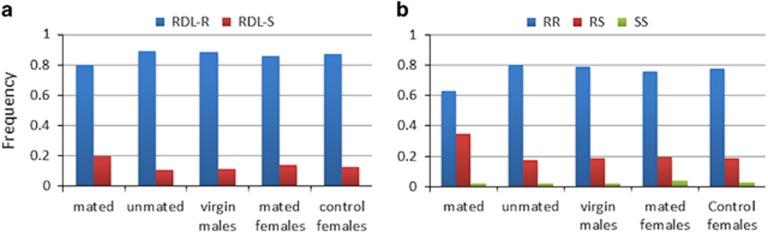
Impact of A296S *RDL* mutation on male mating success. Distribution of *RDL* alleles (**a**) and genotypes (**b**) between coupled and uncoupled males in comparison with control mosquitoes.

A very high frequency of the resistant *RDL*^*R*^ allele was observed in all samples, ranging from 80.2% in coupled males to 89.1% in uncoupled males (Figure 2a). Consequently, only a very low frequency of the homozygote susceptible genotype (SS), ranging from 2.3 to 4.35%, was detected (Table 3). A comparison of the distribution of the genotypes between samples revealed that the coupled males significantly differed from uncoupled males and from the other two samples (Supplementary Table S6; Figure 2b). Mosquitoes that successfully mated had a lower frequency of the homozygote-resistant genotype (296S/S), with 62.8% compared with 80.4% in mosquitoes that did not mate. Heterozygous mosquitoes were more predominant among those that successfully mated, with a frequency of 34% compared with 17.4% in uncoupled males. Overall, heterozygous mosquitoes are significantly more successful in mating than homozygous-resistant RR mosquitoes (OR=2.58; *P*=0.007) (Table 2). The very low frequency of SS mosquitoes did not allow meaningful statistical comparison with other genotypes.

#### Impact of additive *kdr/RDL-*resistant genotypes on mating success

**Figure 3:**
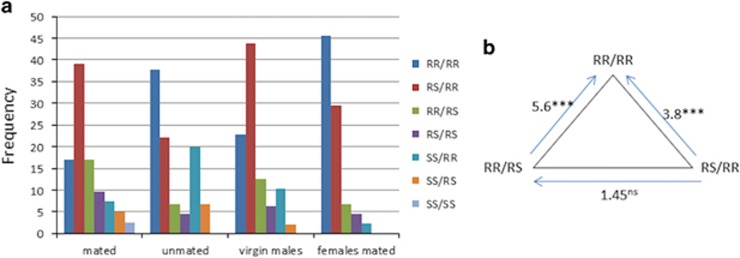
Cumulative impact of both *kdr* and *RDL* mutations on male mating competiveness. (**a**) Distribution of genotype combinations (*kdr/RDL*) between different samples. (**b**) Schematic representation of the impact of some genotype combinations on mating success with odd ratio (OR); *** represents *P*<0.001; NS, not significant.

An attempt was made to assess whether the presence of double resistance alleles for both *kdr* and *RDL* induced a stronger negative impact on mating success. The most predominant genotype combinations observed were the double homozygote resistant (RR/RR), the heterozygote *kdr* and homozygote-resistant *RDL* (RS/RR) and the homozygote-resistant *kdr* and the heterozygote RDL (RR/RS). A higher frequency of double homozygote-resistant genotypes (RR/RR) was observed in mosquitoes that did not mate than in those that mated (37.8 vs 17.1%, respectively). In contrast, mosquitoes with at least a susceptible allele for one of the mutations were predominant among the mated ones (39 vs 22.2% for RS/RR and 17.1 vs 6.7% for RR/RS) (Figure 3a). Consequently, both RR/RS (OR=5.6; *P*<0.001) and RS/RR (OR=3.8; *P*<0.001) mosquitoes displayed a significantly higher advantage in mating compared with the RR/RR double homozygote-resistant mosquitoes (Figure 3b).

#### Impact of metabolic resistance mechanism on mating success

Comparative genome-wide transcription profiling was performed between males that had mated and those that had not, to determine whether the upregulation of metabolic resistance genes affects mating success or not.

### Gene expression profiling associated with mating in male mosquitoes

The direct comparison of the transcription profile between the coupled and uncoupled males provided an opportunity to explore the changes in gene expression associated with mating in male mosquitoes. Owing to the fact that the whole mosquito body was used, with the possibility of a dilution of the level of change for genes expressed in a tissue-specific manner, the list of genes differentially expressed was established using a fold-change cutoff of +/−1.5, instead of the traditional +/−2, with *q*<0.05. A total of 2205 probes were differentially expressed by this criterion (1305 upregulated in mated males vs 900 downregulated).

### Genes upregulated in mated relative to unmated males

**Figure 4:**
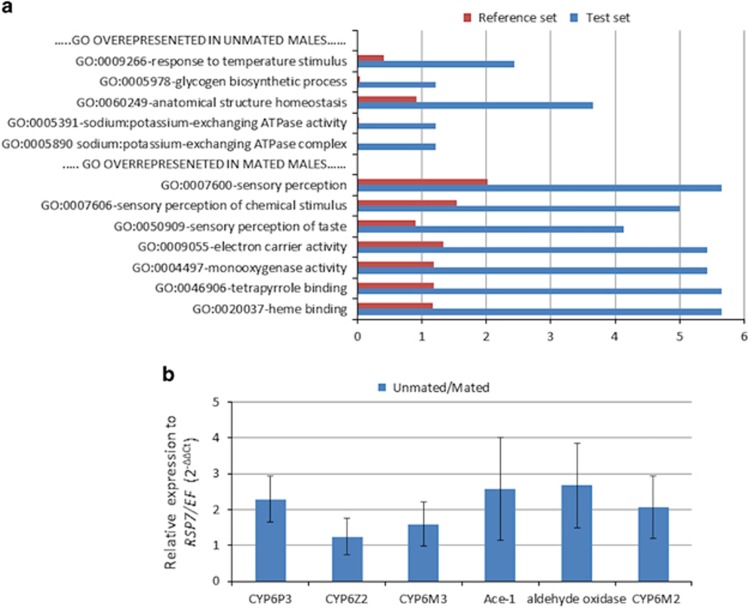
Gene expression profiling. (**a**) Gene ontology (GO) terms enriched in the sets of genes overexpressed in both mated and unmated male mosquitoes in VK. The overrepresented GO terms have been detected using BLAST2GO with the false discovery rate multiple correction test applied at *P*<0.05. (**b**) Differential expression of metabolic resistance genes by qRT-PCR between mated and unmated mosquitoes.

Analysis of the list of genes overexpressed in males that successfully coupled with females revealed a significant enrichment of genes belonging to GO terms of sensory perception, sensory perception of chemical stimulus and sensory perception of taste (Figure 4). This group includes genes coding for gustatory receptors (for example, AGAP001115-RA, AGAP007757-RA), odorant receptors (for example, AGAP004278-RA, AGAP009397-RA), olfactory receptors (for example, AGAP009390-RA, AGAP004974-RA) and also odorant-binding proteins (for example, AGAP002025-RA, AGAP002190-RA). The overexpression of genes belonging to these GO terms could relate to the mating process as males seek to locate and interact with the females.

Other enriched GO terms are related to heme binding and mono-oxygenases activities. This group includes several detoxifying cytochrome P450 genes with *CYP6AD1* being the most overexpressed (FC 2.85). Similar GO enrichment carried out using the program DAVID confirmed the overrepresentation of these gene families but also detected an enrichment of GO terms for peroxidase activity, antioxidant activity and to response to oxidative stress (Supplementary Figure S2A). This group comprises sets of genes coding for chorion peroxidase including PX10, PX11 and PX15. Another set is made of several oxidase peroxidases including PX4B (FC3.6), PX14, PX8, PX9, PX5A and PX3. A similar group of genes regulating oxidative stress including PX15 was recently also found to be upregulated in female spermatheacae in relation to sperm storage (Shaw *et al.*, 2014). In addition, the peroxidase PX4B was also found to be upregulated in females as a consequence of mating (Rogers *et al.*, 2008). Another group of upregulated genes comprises several juvenal hormone responsive genes (*AGAP003220-RA*, *AGAP013267-RA*, *AGAP010765-RA*), which are known to be produced by the male accessory glands and transferred to the female during mating (Rogers *et al.*, 2008).

Several probes belonging to carboxylesterase genes were upregulated in mated males, including *COE2580*, *AGAP001723-RA*, *COE22933*, *AGAP011365-RA* and *COE18026*. Another group of genes significantly enriched included several cuticular protein genes (*AGAP008460-RA*, *AGAP008458-RA*, *AGAP010908-RA*, *AGAP003385-RA*) with the GO term of structural constituent of cuticle significantly enriched in the DAVID analysis.

Among the highly upregulated genes in mated mosquitoes were genes such as the discoidin domain receptor gene (*FC5.9*), which is known to be activated by collagen, the sex-related gene sex-determining region y sry (AGAP010919-RA) (FC 3.6) and many zinc-finger protein genes (for example, *AGAP004575-RA* and *AGAP007515-RA*). A set of immune genes was also upregulated in mated males, including the antimicrobial protein defensin (*AGAP011294-RA*), two thioester-containing protein genes, *TEP1* (AGAP010814-RA) and *TEP9* (AGAP010830-RA), the *APL1C* (AGAP007033-RA) gene and many leucine-rich repeat proteins (AGAP007829-RA, AGAP002575-RB, AGAP011378-RA). Further details are presented in Table 4.

### Genes downregulated in mated relative to unmated males

Enrichment analysis with BLAST2GO revealed an overrepresentation of genes belonging to GO term ‘response to temperature stimulus' including heat-shock protein genes (*AGAP007160-RD*, *AGAP007161-RA*, *AGAP005548-RA*) (Figure 4). Genes belonging to GO terms associated with ATPase activity were also downregulated in mated males as well as genes associated with the glycogen biosynthetic process. In addition, DAVID functional analysis also indicated an enrichment of cytochrome P450 genes including *CYP307B1, CYP307A1* and *CYP325C2* observed among the most downregulated genes (Supplementary Figure S2B). Other downregulated genes included sex-related genes such as two sex-determining fem-1 genes (*AGAP004499-RB*, *AGAP007382-RA*), three testis-specific genes including a testis-specific serine threonine kinase (AGAP008735-RA), a testis-specific protein pbs 13 (AGAP000633-RA) and a testis development protein (AGAP009027-RA). Further details are presented in Supplementary Table S7.

### Comparative analysis of expression profiles in mating and insecticide resistance

To assess the impact of metabolic resistance on the mating success, the mating expression profile was compared with that of insecticide resistance in the VK village to detect possible association between main resistance genes and the mating outcome. The expression profile of insecticide resistance generated by Kwiatkowska *et al.* (2013) from mosquitoes collected from VK, at the same time as this study, was used to detect a set of insecticide resistance genes upregulated or downregulated in mated male mosquitoes in comparison with unmated males. The hypothesis was that if genes upregulated in resistant mosquitoes are found significantly downregulated in mated males it could indicate a detrimental effect on mating competiveness in males. In the opposite case, where these resistance genes are upregulated in mated males, they could be conferring a mating advantage. A Venn-diagram was used to show the sets of genes commonly differentially expressed in mated mosquitoes and in insecticide-resistant mosquitoes.

Analysis of the set of detoxification genes upregulated in mated mosquitoes revealed that only few were also upregulated in resistant mosquitoes. This list (Supplementary Table S8) included the cytochrome P450 genes *CYP6AD1, CYP6P5* and *CYP4H19* and one oxidase peroxidase PX6. In contrast, three detoxification genes were downregulated in resistant mosquitoes including a chorion peroxidase (PX10), a thioredoxin protein (GRX2) and a cytochrome P450 (AGAP009241-RA). The vast majority of detoxification genes upregulated in mated mosquitoes were not associated with insecticide resistance as they were neither upregulated nor downregulated in resistant mosquitoes.

Analysis of the set of detoxification or resistance-related genes downregulated in the mated mosquitoes showed three genes associated with insecticide resistance through overexpression in the resistant VK samples (Supplementary Table S9). This set of genes possibly negatively impacting on male mating success includes an aldehyde dehydrogenase (with two transcripts AGAP005124-RA and RB), a lethal essential for life (AGAP007161-RA) and a cytochrome P450 gene (*CYP6P2*). In contrast, several of these downregulated detoxification genes in mated mosquitoes were also downregulated in resistant mosquitoes including a nitrilase member 2, cytochrome P450s and ABC transporters. Overall, most of the detoxification genes downregulated in mated mosquitoes were not associated with insecticide resistance as they were neither upregulated nor downregulated in resistant mosquitoes.

Specific attention was paid to the expression profile of all of the detoxification (or resistance related) genes upregulated in resistant mosquitoes between mated and uncoupled males (Supplementary Table S10). This analysis revealed that among the 43 genes associated with metabolic resistance in VK, more genes were in fact also upregulated in mated males (16) than downregulated (3) suggesting that metabolic resistance genes may only have a limited impact on mating competiveness. All the main detoxification genes with fold changes above four were not even differentially expressed between mated and unmated males, further supporting that genes such as *CYP6P3*, *CYP6M2, CYP6Z2* or aldehyde oxidase, which have been confirmed as the main insecticide resistance genes in VK, do not have an impact on mating competiveness. The expression pattern of these resistance genes between mated and unmated mosquitoes was further validated by qRT-PCR, with fold-changes comparable between both sets of mosquitoes for *CYP6Z2, CYP6P3, CYP6M2* or aldehyde oxidase (Figure 4b).

## Discussion

The extent to which insecticide resistance impacts on mosquito biological traits remains largely uncharacterized despite the major influence it could have on the implementation of insecticide resistance management strategies to mitigate the damage of resistance to control interventions. This study has investigated the impact of insecticide resistance on the mating competiveness of male *An. gambiae* mosquitoes and also explored the molecular changes associated with mating in these male mosquitoes.

### Mating in *An. gambiae*

As female mosquitoes generally mate only once in their lifetime, mating is a key event in the genetic make-up of a mosquito population as it is most likely to influence the next generation. It is possible that if some male mosquitoes were more able to mate than others, notably in relation to insecticide resistance, this could impact the spread of insecticide resistance genes or alleles in mosquito populations. For this reason, further characterization of mating process in mosquitoes is clearly important in understanding the evolution of insecticide resistance within mosquito populations. In this study, a strict segregation in swarms of the M and S molecular forms of *An. gambiae* was observed, as previously described (Diabate *et al.*, 2006), as all mosquitoes from the swarms were of the M form (now *An. coluzzii*). The mechanisms involved in such segregation include differences in structure, timing and locations of swarms (Sawadogo *et al.*, 2014). Assortative mating previously reported between the two species was also apparent in our collected population from VK3, with solely M form mosquitoes detected in this study, although one should note that *An. coluzzii* was clearly predominant in the village during this season as no *An. gambiae* (S form) was also collected indoor. The absence of the S form (*An. gambiae*) in VK3 is in line with previous reports showing that the form composition of mosquitoes shifts seasonally, with M forms thriving in the dry seasons, as described by Diabate *et al.* (2009).

### Target site resistance negatively impacts mating success

This study has revealed that the presence of target site resistance mechanism affects the mating competiveness of male *An. gambiae* mosquitoes in natural populations. This was seen with the L1014F *kdr* mutation as heterozygote male mosquitoes were significantly more likely to mate than 1014F homozygote-resistant ones suggesting that *kdr* mutation has a detrimental effect on the mating ability of *An. coluzzii* males. More precisely, this suggests that there is a fitness cost associated with possessing double alleles of the 1014F mutation rather than having just one allele. Indeed, the swarms were predominantly composed of homozygote resistant males; however, it is the heterozygote males that are predominantly selected by females for mating. Therefore, the increase in the frequency of *kdr* allele observed in the past decade in the M form in VK can be attributed to the selection pressures imposed by the intensive use of insecticides, rather than to any increased reproductive success of homozygote-resistant individuals.

This reduced mating success in homozygote RR *kdr* males should contribute to slow the speed of increase of the frequency of this resistance allele in the wild and will also prevent or delay the fixation the *kdr*^*R*^ allele in the population. The reduced mating success of homozygote RR *kdr* male *An. coluzzii* could facilitate the implementation of resistance management strategies such as rotation of insecticides. Indeed the removal of the selection pressure from pyrethroids by using a different insecticide class such as carbamates could allow a reversal to susceptibility with the decrease in the frequency of RR-Kdr mosquitoes in the populations. Such an effect has previously been observed in a population of *An. darlingi* resistant to pyrethroid in Columbia, where the removal of pyrethroid insecticides led to the recovery of susceptibility 4 years later (WHO, 2012).

The reduced mating success of RR-kdr males could be explained by the significant impact that such a change on the voltage-gated sodium channel may induce on the neural network, potentially affecting many physiological traits in resistant mosquitoes including mobility, perception of stimuli or even the olfactory system (Rivero *et al.*, 2010). Such effects are possibly supported by the microarray results in this study, which show a significant enrichment of GO terms associated with perception of stimuli or perception of taste in males that successfully mated compared with those that did not. It is possible that possessing double alleles of the *kdr-*resistant mutation could prevent these male *An. coluzzii* mosquitoes from quickly detecting females entering the swarm, in contrast to the heterozygote males. This possibility is supported by previous observations in peach-potato aphids, for which *kdr*-resistant individuals were found to have a reduced excitability of their nervous system and as a consequence they were less responsive to the presence of pheromone released by other aphids (Foster *et al.*, 2003; Rivero *et al.*, 2010). This could also be the case in the homozygote RR-kdr males in *An. coluzzii* that could be less responsive to olfaction cues from females than the heterozygote males. Indeed, sodium channels are implicated in olfactory signal transduction from the olfactory receptors to the central nervous system (Zwiebel and Takken, 2004). The downregulation of GO terms associated with olfaction in uncoupled mosquitoes support this hypothesis. In addition, it was shown that the *kdr-*resistant aphids were less responsive to change in temperature gradient than susceptible ones (Rivero *et al.*, 2010). A similar situation in the RR-kdr-resistant *An. gambiae* males could explain their reduced mating success because of their inability to respond properly and/or as quickly as the heterozygote males to all the stimuli involved during the coupling process in a swarm. Further studies are clearly needed to establish the factors responsible for the advantage of heterozygote RS *kdr* male *An. coluzzii* mosquitoes.

Interestingly, it was also observed that heterozygote males had a fitness advantage over the homozygote susceptible ones. The fact that heterozygote males have a higher mating success than both resistant and susceptible homozygotes suggests the possible existence of a heterozygote advantage effect for *kdr* in *An. gambiae*. If such heterozygote advantage was confirmed, it could make it difficult to completely remove the resistance allele from the population even after switching from pyrethroids to other insecticide classes not targeting the sodium channel. This should be further investigated, particularly to see whether such heterozygote advantage also extends to other fitness traits such as fecundity or longevity.

Analysis of the distribution of *RDL* genotypes between mated and unmated males revealed a similar pattern than for *kdr* with a fitness advantage for the heterozygote 296A/S, which had a higher mating success than the homozygote resistant 296S/S males. Because the *RDL* mutation in the GABA receptor gene also influences the neural network of the mosquito (Rivero *et al.*, 2010), it is likely that the same causes that induce the reduced mating success of homozygote *kdr* RR are also those acting on *RDL-*resistant homozygote. Possession of two *RDL* resistance alleles could therefore also impact negatively of the ability of these males to perceive external stimuli (olfaction, visual) or even make those sluggish compared with heterozygotes. This result is in accordance with a previous study assessing the impact of dieldrin resistance on mating behavior of both male and female *An. gambiae* laboratory strains, which demonstrated a fitness disadvantage in both sexes: the females exhibiting significantly decreased fecundity and a slower response to predators while resistant males had fewer successful copulations (Rowland, 1991a, b). The frequency of homozygote *RDL* susceptible mosquitoes was too low in VK3 to assess whether the heterozygote males were also more likely to mate than susceptible SS as seen for *kdr*. It remains also to explain why the frequency of the *RDL* allele remains high in this population despite the fact that dieldrin insecticide is no longer used in the public health sector. The reduced mating success of homozygote resistant should normally have contributed in reducing the frequency of the allele in the population if the selection from dieldrin or similar cyclodiene insecticide was absent. Therefore, it is possible that such selection could still be ongoing but from the agricultural sector as observed in La Reunion (Tantely *et al.*, 2010). This is further corroborated by the maintenance of similar high frequency of *RDL*^*R*^ allele in the other malaria vector *An. funestus* across West and central Africa (Wondji *et al.*, 2011).

The impact of target site resistance mechanisms on the mating success of male *An. coluzzii* was further highlighted by the presence of an additive fitness cost in the homozygote-resistant males for both *kdr* and *RDL* mutations. This increased fitness cost when a male mosquito is homozygous for resistance alleles for both mutations could be caused by a cumulative negative impact on the neural network affecting the ability to detect external stimuli, for example. The detection of such additive fitness cost suggests that the overall fitness cost of a specific target site mutation should also be assessed by taking into consideration its interaction with other target site mutations in the population. In this regard, although dieldrin is no more recommended in the public health sector, it could still be very relevant to detect the *RDL* mutation in field populations to understand the impact of other target site mutations such as *kdr* and *Ace-1*.

In contrast to *kdr* and *RDL*, analysis of the frequency of resistant *Ace-1* alleles did not detect an impact on mating competitiveness of resistant males. However, the absence of any homozygote resistant allele in this study is a suggestion of the possible fitness cost associated with this allele as previously reported in other populations (Djogbenou *et al.*, 2010). A previous study in *C. pipiens* to assess the impact of homozygote-resistant *Ace-1*^*R*^ genotype on mating suggested a detrimental effect of this resistance allele on competitive mating ability of males compared with susceptible males (Berticat *et al.*, 2002). It would be interesting to assess whether the same effect is seen in *An. gambiae* individuals from a field population with a higher frequency of the *Ace-1*^*R*^ allele than in VK.

### Metabolic resistance has limited fitness cost on mating success

In contrast to the target site resistance mechanism, metabolic resistance was not found to significantly influence the mating competitiveness of male *An. coluzzii* mosquitoes. First, most of the detoxification genes associated with resistance to insecticides in VK such as *CYP6P3, CYP6Z2, CYP6M2* and aldehyde oxidase (Kwiatkowska *et al.*, 2013) were not differentially expressed between the mated and unmated males suggesting that overexpression of these genes does not impact mating success. However, a P450, *CYP6P2*, overexpressed in resistant mosquitoes was found to be downregulated in males that mated suggesting that, although not common, some metabolic resistance genes could still be associated with a negative impact on mating competiveness. Second, the fact that 16 out of 19 resistance genes upregulated in resistant mosquitoes in VK and also differentially expressed between mated and unmated mosquitoes were rather upregulated in mated males further suggests that overexpression of detoxification genes does not provide a fitness disadvantage to male *An. coluzzii* mosquitoes, at least in terms of mating success. This is also supported by the enrichment of GO terms associated with peroxidase and cytochrome P450s in mated mosquitoes. The lack of fitness cost associated with metabolic resistance in this study could be explained by the fact that metabolic resistance genes belong to large gene families with broad catalytic activities and therefore are unlikely to be adversely affected by mutations in the same way as specific target site mutations affect genes such as the sodium channel, with vital functions and the need for a highly conserved genomic sequence. Therefore, metabolic resistance, notably through over-expression as found for the VK population, can confer a high level of protection against insecticides without significant fitness cost to the resistant mosquitoes in relation to mating. However, it has previously been reported that other metabolic resistance mechanisms such as the overproduction of carboxylesterases could confer a significant fitness cost as observed in resistant *C. pipiens*, which shows a reduced locomotive performance than susceptible ones. It was suggested that such reduced performance was caused by a resource depletion linked to the overproduction of carboxylesterases (Berticat *et al.*, 2004). However, such overproduction of a specific gene was not observed in VK as the fold change of detoxification genes was not too high, with the highest being for the P450 *CYP6Z2* genes at FC20.5.

## Conclusion

Understanding the impact of insecticide resistance on various biological and behavioral traits of malaria vectors is an important prerequisite to improve the effectiveness and success of current and future malaria insecticide-based vector control interventions. The present study has revealed for the first time in field populations that insecticide resistance through target site resistance mechanisms exerts a fitness cost on mating competiveness of wild male *Anopheles* mosquitoes. Because such negative fitness costs could influence the evolution of insecticide resistance in field populations of mosquitoes, such as the speed of increase or reversal of frequency of resistance alleles in a population, it is imperative that such impacts are understood and taken into consideration when designing and implementing future insecticide resistance management strategies.

## Data archiving

The microarray data from this study are submitted to Array Express accession number E-MTAB-3314.

## Figures and Tables

**Table 1:** Genotype distribution of the L1014F *kdr* mutation in relation to mating success

*Sample group*	*Number successfully genotyped*	*Genotypes (%)*				
		*1014F*	*L1014*	*TTA*	*TTA/T*	*TTT*
		*R*	*S*	*SS*	*SR*	*RR*
Mated males	45	61.1	38.9	7 (15.6)	21 (46.7)	17 (37.8)
Uncoupled males	48	60.5	39.5	13 (27.1)	12 (25.0)	23 (47.9)
F1 virgin males	49	60.2	39.8	7 (14.3)	25 (51.0)	17 (34.7)
Mated females	47	74.4	25.5	2 (4.3)	20 (42.6)	25 (53.2)
Total	189	64.1	35.9	29 (15.3)	78 (41.3)	82 (43.4)

Abbreviations: RR, homozygous resistant; SR, susceptible resistant heterozygous; SS, homozygous susceptible. Numbers in parentheses indicate the relative frequency in each sample group.

**Table 2:** Assessment of the association of different genotypes at target site mutations with mating success

*Genotypes*	*kdr*		*RDL*		*Ace-1*	
	*Odds ratio*	P *value*	*Odds ratio*	P *value*	*Odds ratio*	P *value*
RR vs RS	2.36 (1.24–4.52)	0.001	2.58 (2.3–4.9)	0.007	/	/
RR vs SS	0.74 (0.11–4.8)	NS	1.33 (0.2–8.7)^a^	NS	/	/
RS vs SS	3.26 (1.47–7.1)	0.006	0.5^a^	NS	0.72 (0.28–1.81)	NS

a Very low number of SS; for odds ratio, confidence interval at 95% are given in brackets; NS, not significant.

**Table 3:** Genotype distribution of RDL mutations in relation to mating success

*Sample group*	*Number successfully genotyped*	*Genotypes (%)*				
		*296S*	*A296*	*GCT*	*G/TCT*	*TCT*
		*R*	*S*	*SS*	*SR*	*RR*
Mated males	43	80.2	19.8	1 (2.3)	15 (34.9)	27 (62.8)
Uncoupled males	46	89.1	10.9	1 (2.2)	8 (17.4)	37 (80.4)
F1 virgin males	48	88.5	11.5	1 (2.1)	9 (18.75)	38 (79.2)
Mated females	46	85.9	14.1	2 (4.3)	9 (19.6)	35 (77.1)
Total	183	86.1	13.9	5 (2.7)	41 (22.4)	137 (74.9)

Abbreviations: RR, homozygous resistant; SR, susceptible resistant heterozygous; SS, homozygous susceptible.

**Table 4:** Top 50 probes upregulated in mated males in comparison with insecticide resistance profiling

*Probes*	*Transcript ID*	*Fold change*	*Description*	*Upregulated in resistant*	*Downregulated in resistant*
CUST_11984_PI422575199	AGAP008743-RA	5.9	Discoidin domain receptor	5.6	
CUST_7119_PI422575199	AGAP004518-RA	3.9	Potassium-dependent sodium-calcium exchanger	4.6	
CUST_3577_PI422575199	AGAP001987-RA	3.8	Peptidyl-prolyl cis-trans isomerase	5.6	
CUST_11419_PI422575199	AGAP008133-RA	3.2	Clavesin-2	10.3	
CUST_13410_PI422575199	AGAP010185-RA	2.7	Echinoid	5.8	
CUST_2074_PI422575199	AGAP005766-RA	2.6	Hexamerin a	2.9	
CUST_5142_PI422575199	AGAP003076-RB	2.6	Pyrokinin receptor	2.0	
CUST_2304_PI422575199	AGAP006792-RA	2.4	AGAP006792-PA (Anopheles gambiae str. PEST)	9.6	
CUST_11005_PI422575199	AGAP010899-RA	2.3	Oxidase peroxidase	6.6	
CUST_9457_PI422575199	AGAP010820-RA	2.3	Serine protease nudel	2.0	
CUST_11889_PI422575199	AGAP008646-RA	2.3	CAMP CGMP cyclic nucleotide phosphodiesterase	2.7	
CUST_3225_PI422575199	AGAP007663-RA	5.7	27 kDa hemolymph protein		7.8
CUST_2470_PI422575199	AGAP006946-RA	4.6	Prefoldin subunit 4		7.1
CUST_2309_PI422575199	AGAP006796-RA	4.1	Peritrophin a		2.7
CUST_11449_PI422575199	AGAP008163-RA	3.3	Nhp2-like protein 1-like		2.3
CUST_2656_PI422575199	AGAP007125-RA	2.7	Wd-repeat protein		2.9
CUST_543_PI422575199	AGAP005149-RA	2.6	H aca ribonucleoprotein complex subunit 3		3.0
CUST_13193_PI422575199	AGAP009968-RA	2.3	Ribosome production factor 1		3.4
CUST_2981_PI422575199	AGAP007420-RA	5.5	Peptidylglycine alpha-hydroxylating monooxygenase		
CUST_12878_PI422575199	AGAP009656-RA	5.4	Zinc-finger protein 3		
CUST_7919_PI422575199	AGAP000718-RA	5.1	Monocarboxylate transporter		
CUST_3655_PI422575199	AGAP002040-RB	5.1	Cell adhesion molecule		
CUST_7208_PI422575199	AGAP004575-RA	4.7	Zinc-finger protein 425		
CUST_1279_PI422575199	AGAP005812-RA	4.4	Hypothetical conserved protein		
CUST_5960_PI422575199	AGAP013255-RA	4.1	Aminopeptidase n		
CUST_3108_PI422575199	AGAP007558-RA	3.8	Major allergen bla g		
CUST_11796_PI422575199	AGAP008534-RA	3.7	Cyclin-dependent kinase 5 activator		
DETOX_731_PI422610884	PX4B	3.6	Oxidase peroxidase		
CUST_1649_PI422575199	AGAP006151-RA	3.6	AGAP006151-PA (Anopheles gambiae str. PEST)		
CUST_3067_PI422575199	AGAP007520-RA	3.6	Peroxisomal membrane protein pmp34		
CUST_9549_PI422575199	AGAP010919-RA	3.6	Sex-determining region y sry		
CUST_5673_PI422575199	AGAP003496-RA	3.6	Adam (a disintegrin and metalloprotease)		
CUST_1340_PI422575199	AGAP005871-RA	3.3	Ribosome biogenesis protein		
CUST_9363_PI422575199	AGAP010719-RA	3.1	Coatomer subunit delta		
CUST_13441_PI422575199	AGAP010217-RA	3.0	Protein disulfide isomerase		
CUST_6512_PI422575199	AGAP013329-RA	2.9	AGAP013329-PA (Anopheles gambiae str. PEST)		
DETOX_416_PI422610884	CYP6AD1	2.9	Cytochrome p450		
CUST_5650_PI422575199	AGAP013065-RA	2.7	Tal-like protein aa		
CUST_11311_PI422575199	AGAP008016-RA	2.6	Acyl- oxidase		
CUST_12102_PI422575199	AGAP008870-RA	2.6	Upf0704 protein c6orf165 homolog		
CUST_12680_PI422575199	AGAP009461-RA	2.6	Stress-activated protein kinase jnk		
CUST_13173_PI422575199	AGAP009948-RA	2.6	Signal recognition particle 19 kda protein		
CUST_9051_PI422575199	AGAP010383-RA	2.6	Oligopeptide transporter		
CUST_5345_PI422575199	AGAP003220-RA	2.5	Juvenile hormone-inducible		
CUST_2156_PI422575199	AGAP006653-RB	2.5	Protein rogdi		
CUST_9785_PI422575199	AGAP011171-RA	2.5	Ribonuclease 29kDa-subunit		
CUST_7047_PI422575199	AGAP004448-RB	2.4	Segment polarity protein disheveled		
CUST_10419_PI422575199	AGAP011842-RA	2.4	Signal peptidase complex subunit 2		
DETOX_259_PI422610884	CYP307B1	2.4	Cytochrome p450		
CUST_11012_PI422575199	AGAP010815-RA	2.4	Tep1		
CUST_9902_PI422575199	AGAP011305-RA	2.3	Alkaline phosphatase		
CUST_1837_PI422575199	AGAP006366-RA	2.3	2-Oxoglutarate dehydrogenase		
CUST_8761_PI422575199	AGAP000397-RA	2.2	Lethal 07882		
