# Supplementary material for: Target-site resistance mutations (kdr and RDL), but not metabolic resistance, negatively impact male mating competiveness in the malaria vector Anopheles gambiae
Source: Heredity (Edinb). 2015 Apr 22;115(3):243–52. doi: 10.1038/hdy.2015.33 (PMC4519523; doi:10.1038/hdy.2015.33)
Supplement: Supplementary Figures [file hdy201533x1.pdf]

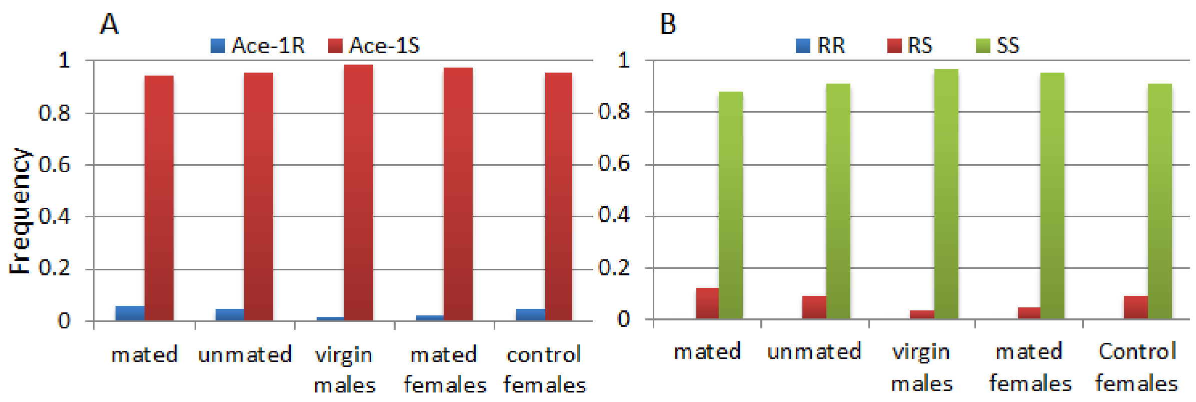

Figure S1: Impact of *Ace-1* mutation on mating success: Distribution of *Ace-1* alleles (A) and genotypes (B) between coupled and uncoupled males in comparison to control mosquitoes.

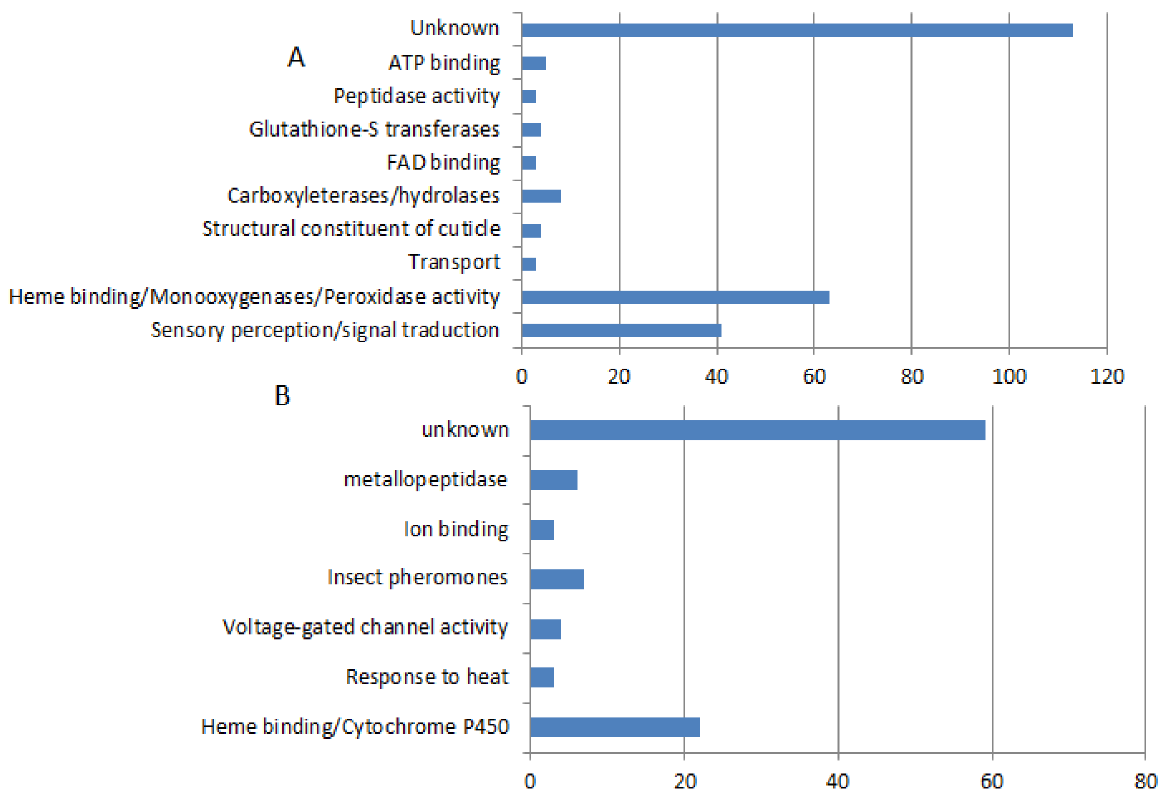

Figure S2: Functional annotation clustering of the classes of genes enriched among the genes up-regulated in mated (A) and unmated (B) males in VK. The DAVID software was used to define these gene groups enriched with Fisher's exact test applied to determine the *P* values.
