# Supplementary material for: Target-site resistance mutations (kdr and RDL), but not metabolic resistance, negatively impact male mating competiveness in the malaria vector Anopheles gambiae
Source: Heredity (Edinb). 2015 Apr 22;115(3):243–52. doi: 10.1038/hdy.2015.33 (PMC4519523; doi:10.1038/hdy.2015.33)
Supplement: Supplementary Tables [file hdy201533x2.doc]

**Table S1: Primers used for qRT-PCR**

| **Genes** | **Forward Primer** | **Reverse Primer** | **Expected size (bp)** |
| --- | --- | --- | --- |
| Aldehyde oxidase | GATATTCCGGTCGACTTTCG | AACGACACGGTCATGTTGAG | 110 |
| CYP6P3 | AGCGATGCTTTGTTTTGCTT | AGCGATGCTTTGTTTTGCTT | 131 |
| CYP6Z2 | AGTTCAAGTTCCAGGCCACA | TTTAGTTCATCACAATCAGTTGC | 138 |
| CYP6M2 | TACGATGACAACAAGGGCAAG | GCGATCGTGGAAGTACTGG | 130 |
| Acetylcholinesterase 1 (Ace-1) | GTTGCAGCTACCTCGAACCT | ACACCAGCAGCACGATCA | 100 |
| CYP6M3 | TGGACCAGATACTGAAGGAGAGT | ACAGAGGTTCCTGCTTCGAG | 121 |
| RSP7 (AGAP010592) | GTGTTCGGTTCCAAGGTGAT | TCCGAGTTCATTTCCAGCTC | 98 |
| Elongation Factor (AGAP005128) | GGCAAGAGGCATAACGATCAATGCG | GTCCATCTGCGACGCTCCGG | 208 |

**Table S2:** Global positioning signal co-ordinates for each swarm location used for collection in VK3, Vallée du Kou. These mating swarms were the sites of couple and uncoupled male collections, on ten occasions in April 2010.

| **Reference** | **Swarm** | **Co-ordinates** | **GPS ref** |
| --- | --- | --- | --- |
| E1 | Pigsty | N 11.37276° W 004.40309 | Ess 1 VK3 |
| E2 | Rubbish heap | N 11.37279° W 004.40360 | Ess 2 VK3 |
| E3 | Waste water (toilet) drainage | N 11.37279° W 004.40364 | Ess 3 VK3 |
| E4 | Drainage, near well | N 11.37277° W 004.40445 | Ess 4 VK3 |
| E5 | Above firewood | N 11.37268° W 004.40442 | Ess 5 VK3 |
| E6 | Close to firewood | N 11.37314° W 004.40513 | Ess 6 VK3 |
| E7 | Well drainage, close to rubbish heap | N 11.37305° W 004.40554 | Ess 7 VK3 |
| E8 | Pigsty | N 11.37313° W 004.40506 | Ess 8 VK3 |

**Table S3: Mating swarm collection details.** Swarms were observed for the entirety of their duration on ten separate occasions in VK3, all couples captured and, following the cessation of copulations, uncoupled males from the swarms also collected.

| Date | Swarm | Number of couples collected | Number of uncoupled males collected | Number of uncoupled females |
| --- | --- | --- | --- | --- |
| 17.4.10 | E1 | 6 | 30 |  |
|  | E2 | 2 | 30 |  |
|  | E3 | 1 | 0 |  |
|  | **Total** | **9** | **60** |  |
| 18.4.10 | E3 | 1 | 10 |  |
|  | **Total** | **1** | **10** |  |
| 22.4.10 | E1 | 1 | 30 |  |
|  | E4 | 3 | 24 |  |
|  | **Total** | **4** | **54** |  |
| 23.4.10 | E1 | 3 | 0 |  |
|  | E3 | 1 | 6 |  |
|  | E5 | 3 | 23 |  |
|  | **Total** | **7** | **29** |  |
| 26.4.10 | E5 | 9 | 73 | 1 |
|  | **Total** | **9** | **73** | **1** |
| 27.4.10 | E5 | 3 | 55 |  |
|  | **Total** | **3** | **55** |  |
| 28.4.10 | E1 | 1 | 22 |  |
|  | E5 | 3 | 30 |  |
|  | **Total** | **4** | **52** |  |
| 29.4.10 | E1 | 2 | 41 |  |
|  | E3 | 11 | 50 | 1 |
|  | E5 | 21 | 60 |  |
|  | E6 | 6 | 10 |  |
|  | E7 | 3 | 14 |  |
|  | **Total** | **43** | **175** | **1** |
| 30.4.10 | E1 | 2 | 17 |  |
|  | E3 | 7 | 30 |  |
|  | E5 | 18 | 20 |  |
|  | E6 | 3 | 20 |  |
|  | E8 | 3 | 20 |  |
|  | **Total** | **33** | **107** |  |

**Table S4:** Insecticide resistance profile for *Anopheles coluzzii* from VK3 in April 2010

| **Insecticide** | **Total number exposed, VK3** | **24 hour mortality (%)** | **Total number exposed, Kisumu** | **Kisumu mortality (%)** |
| --- | --- | --- | --- | --- |
| 0.75% permethrin | 99 | 6 | 100 | 100 |
| 4% DDT | 100 | 12 | 101 | 100 |
| 0.1% bendiocarb | 101 | 91 | 100 | 100 |

**Table S5:** Comparison of *kdr* genotype distribution between the mated and uncoupled samples using a chi-square analysis

| Sample group | Chi squared (p value) | | |
| --- | --- | --- | --- |
|  | F1 virgin males | Uncoupled males | Mated females |
| Mated males | 0.759 (0.684) | 25.8 (<0.001) | 34.8 (<0.001) |
| Mated females | 18.3 (<0.001) | 130.0 (<0.001) | - |
| Uncoupled males | 29.8 (<0.001) | - | - |

**Table S6:** Comparison of *RDL* genotype distribution between the mated and uncoupled samples using a chi-square analysis

| Sample group | Chi squared (p value) | | |
| --- | --- | --- | --- |
|  | F1 virgin males | Uncoupled males | Mated females |
| Mated males | 17.3 (0.000) | 21.5 (0.000) | 15.2 (0.001) |
| Mated females | 1.28 (0.526) | 1.52 (0.469) |  |
| Uncoupled males | 0.127 (0.939) |  |  |

**Table S7**: Top 50 most down-regulated probes in mated mosquitoes

| **Probes** | **Transcript ID** | **Fold Change** | **Description** | **Up in R** | **Down in R** |
| --- | --- | --- | --- | --- | --- |
| CUST_3544_PI422575199 | AGAP001970-RA | 4.1 | ubiquitin c | 2.3 |  |
| CUST_4143_PI422575199 | AGAP002364-RA | 3.6 | immt_drome ame: full= mitochondrial inner membrane protein | 13.5 |  |
| CUST_9667_PI422575199 | AGAP011052-RA | 3.4 | aldo-keto reductase | 2.0 |  |
| CUST_4142_PI422575199 | AGAP002364-RB | 3.1 | immt_drome ame: full= mitochondrial inner membrane protein | 13.5 |  |
| CUST_11714_PI422575199 | AGAP008450-RA | 3.9 | AGAP008450-PA [Anopheles gambiae str. PEST] |  | 6.8 |
| CUST_236_PI422575199 | AGAP004880-RC | 3.7 | l-lactate dehydrogenase |  | 8.9 |
| CUST_237_PI422575199 | AGAP004880-RA | 3.6 | l-lactate dehydrogenase |  | 9.7 |
| CUST_7412_PI422575199 | AGAP001603-RA | 3.1 | mitochondrial 18 kda protein |  | 2.3 |
| CUST_10357_PI422575199 | AGAP011775-RA | 3.0 | phd finger protein |  | 4.8 |
| CUST_3176_PI422575199 | AGAP007621-RB | 2.9 | cytochrome c oxidase subunit viic |  | 5.0 |
| CUST_9664_PI422575199 | AGAP011049-RA | 2.9 | isoform b |  | 2.1 |
| CUST_7849_PI422575199 | AGAP000675-RB | 2.7 | isoform b |  | 2.5 |
| CUST_13008_PI422575199 | AGAP009790-RA | 2.7 | chitin binding peritrophin- |  | 2.4 |
| CUST_11247_PI422575199 | AGAP007952-RA | 2.6 | ubiquitin-conjugating enzyme e2-24 kda |  | 5.9 |
| CUST_8855_PI422575199 | AGAP000469-RB | 2.6 | uncharacterized protein c6orf106-like protein |  | 3.0 |
| DETOX_689_PI422610884 | NIT8537 | 2.6 | nitrilase member 2 |  | 2.4 |
| CUST_7076_PI422575199 | AGAP004458-RC | 3.5 | internalin a |  |  |
| CUST_11086_PI422575199 | AGAP007791-RA | 3.4 | sodium potassium-dependent atpase beta-2 subunit |  |  |
| CUST_11711_PI422575199 | AGAP008447-RA | 3.4 | AGAP008447-PA [Anopheles gambiae str. PEST] |  |  |
| CUST_4613_PI422575199 | AGAP002704-RA | 3.4 | AGAP002704-PA [Anopheles gambiae str. PEST] |  |  |
| CUST_5447_PI422575199 | AGAP003308-RA | 3.3 | isoform b |  |  |
| CUST_4268_PI422575199 | AGAP002456-RB | 3.3 | sarcalumenin |  |  |
| DETOX_210_PI422610884 | CPLC8 | 3.2 | AGAP008446-PA [Anopheles gambiae str. PEST] |  |  |
| CUST_7962_PI422575199 | AGAP013076-RA | 3.2 | AGAP013076-PA [Anopheles gambiae str. PEST] |  |  |
| CUST_4176_PI422575199 | AGAP013481-RE | 3.2 | isoform e |  |  |
| CUST_4449_PI422575199 | AGAP002613-RA | 3.1 | pupal cuticle protein |  |  |
| DETOX_209_PI422610884 | CPLC8 | 3.1 | AGAP008446-PA [Anopheles gambiae str. PEST] |  |  |
| CUST_12317_PI422575199 | AGAP009084-RA | 3.1 | sjchgc08782 protein |  |  |
| CUST_8049_PI422575199 | AGAP001493-RA | 3.1 | pox meso |  |  |
| CUST_10443_PI422575199 | AGAP011879-RA | 3.1 | uncharacterized protein c16orf68 |  |  |
| CUST_10125_PI422575199 | AGAP011530-RA | 3.0 | protein isoform a-like |  |  |
| CUST_4538_PI422575199 | AGAP001293-RA | 3.0 | coiled-coil domain-containing protein 39 |  |  |
| CUST_8352_PI422575199 | AGAP001039-RB | 2.9 | cytochrome p450 |  |  |
| CUST_6625_PI422575199 | AGAP004160-RA | 2.9 | isoform d |  |  |
| CUST_6113_PI422575199 | AGAP003794-RB | 2.9 | hrp65 protein |  |  |
| CUST_5260_PI422575199 | AGAP003161-RA | 2.9 | AGAP003161-PA [Anopheles gambiae str. PEST] |  |  |
| CUST_4269_PI422575199 | AGAP002456-RA | 2.8 | sarcalumenin |  |  |
| CUST_3959_PI422575199 | AGAP002245-RA | 2.8 | mitochondrial ubiquinol-cytochrome c reductase hinge protein |  |  |
| CUST_4066_PI422575199 | AGAP001101-RA | 2.8 | juvenile hormone esterase |  |  |
| CUST_10362_PI422575199 | AGAP011779-RA | 2.8 | AGAP011779-PA [Anopheles gambiae str. PEST] |  |  |
| CUST_5497_PI422575199 | AGAP003341-RB | 2.8 | ecotropic viral integration site |  |  |
| CUST_12781_PI422575199 | AGAP009561-RA | 2.8 | transcription factor castor (protein ming) |  |  |
| CUST_12772_PI422575199 | AGAP009552-RA | 2.7 | sulfotransferase |  |  |
| CUST_4765_PI422575199 | AGAP002816-RA | 2.7 | endoplasmic reticulum oxidoreductin-1-like |  |  |
| CUST_12551_PI422575199 | AGAP009330-RA | 2.7 | troponin c |  |  |
| CUST_4858_PI422575199 | AGAP013202-RA | 2.7 | AGAP013202-PA [Anopheles gambiae str. PEST] |  |  |
| CUST_8385_PI422575199 | AGAP000128-RA | 2.7 | synaptic vesicle protein |  |  |
| CUST_276_PI422575199 | AGAP004914-RA | 2.7 | beta 4 |  |  |
| DETOX_256_PI422610884 | CYP307A1 | 2.6 | cytochrome p450 |  |  |
| CUST_246_PI422575199 | AGAP004888-RA | 2.6 | uncharacterized protein kiaa1841-like protein |  |  |

**Table S8**: Detoxification (or insecticide resistance related) genes over-expressed in mated male mosquitoes in VK

|  | |  | | **Mating** | | | | **Resistance to insecticides** | | | |
| --- | --- | --- | --- | --- | --- | --- | --- | --- | --- | --- | --- |
|  |  | | FC | |  | | FC up | | FC down | |  |
| CUST_11005_PI422575199 | AGAP010899-RA | | 2.3 | | oxidase peroxidase, PX6 | | 6.6 | |  | |  |
| DETOX_86_PI422610884 | COE2580 | | 1.8 | | carboxylesterase | | 4.5 | |  | |  |
| CUST_11216_PI422575199 | AGAP007920-RA | | 1.8 | | glucosyl glucuronosyl transferases | | 2.4 | |  | |  |
| DETOX_417_PI422610884 | CYP6AD1 | | 1.8 | | cytochrome p450 | | 2.3 | |  | |  |
| CUST_4820_PI422575199 | AGAP002866-RA | | 1.8 | | cytochrome p450, CYP6P5 | | 3.1 | |  | |  |
| CUST_7895_PI422575199 | AGAP000088-RA | | 1.7 | | cytochrome p450, CYP4H19 | | 4.3 | |  | |  |
| DETOX_697_PI422610884 | PX10 | | 2.1 | | chorion peroxidase | |  | | 4.6 | |  |
| DETOX_561_PI422610884 | GRX2 | | 2.1 | | thioredoxin domain-containing protein 17-like | | | | 2.1 | |  |
| CUST_12467_PI422575199 | | AGAP009241-RA | | 1.6 | | cytochrome p450 | |  | | 2.6 |  |
| **Top most up-regulated detoxification genes in mated not differentially expressed in resistant mosquitoes** | | | | | | | | | | |  |
| DETOX_731_PI422610884 | PX4B | | 3.6 | | oxidase peroxidase | |  | |  | |  |
| DETOX_416_PI422610884 | CYP6AD1 | | 2.9 | | cytochrome p450 | |  | |  | |  |
| DETOX_259_PI422610884 | CYP307B1 | | 2.4 | | cytochrome p450 | |  | |  | |  |
| DETOX_802_PI422610884 | TPX2 | | 2.2 | | prx- prx- prx-3 | |  | |  | |  |
| CUST_2382_PI422575199 | AGAP006868-RB | | 2.1 | | cuticular protein rr-2 family (agap006868-pa) | | | |  | |  |
| CUST_9583_PI422575199 | AGAP010966-RA | | 1.9 | | cytochrome p450 | |  | |  | |  |
| DETOX_712_PI422610884 | PX14 | | 1.9 | | oxidase peroxidase | |  | |  | |  |
| DETOX_185_PI422610884 | COEBE4C | | 1.8 | | esterase fe4 | |  | |  | |  |
| DETOX_825_PI422610884 | TRXR | | 1.8 | | thioredoxin reductase | |  | |  | |  |
| DETOX_747_PI422610884 | PX8 | | 1.8 | | oxidase peroxidase | |  | |  | |  |
| CUST_12368_PI422575199 | AGAP009137-RA | | 1.8 | | glucosyl glucuronosyl transferases | |  | |  | |  |
| DETOX_515_PI422610884 | CYP9L3 | | 1.7 | | cytochrome p450 | |  | |  | |  |
| CUST_3897_PI422575199 | AGAP002209-RA | | 1.7 | | cytochrome p450 | |  | |  | |  |
| DETOX_405_PI422610884 | CYP4J9 | | 1.7 | | cytochrome p450 | |  | |  | |  |
| DETOX_560_PI422610884 | GRX2 | | 1.7 | | thioredoxin domain-containing protein 17-like | | | |  | |  |
| DETOX_716_PI422610884 | PX15 | | 1.7 | | chorion peroxidase | |  | |  | |  |
| DETOX_458_PI422610884 | CYP6P2 | | 1.7 | | cytochrome p450 | |  | |  | |  |

**Table S9:** Detoxification genes (or insecticide resistance related) down-regulated in uncoupled male mosquitoes in VK

|  |  | **Mating** | | **Resistance to insecticides** | | |
| --- | --- | --- | --- | --- | --- | --- |
|  |  | **FC** | | **FC up** | **FC down** | |
| CUST_514_PI422575199 | AGAP005124-RA | 2.4 | aldehyde dehydrogenase | 2.1 |  | |
| CUST_2696_PI422575199 | AGAP007161-RA | 2.0 | lethal essential for life l2efl | 8.5 |  | |
| CUST_515_PI422575199 | AGAP005124-RB | 1.9 | aldehyde dehydrogenase | 2.8 |  | |
| CUST_4860_PI422575199 | AGAP002869-RA | 1.8 | cytochrome p450, CYP6P2 | 2.2 |  | |
| DETOX_689_PI422610884 | NIT8537 | 2.6 | nitrilase member 2 |  | 2.4 | |
| CUST_1115_PI422575199 | AGAP005656-RA | 2.4 | cytochrome p450 |  | 2.9 | |
| CUST_2695_PI422575199 | AGAP007160-RD | 2.1 | lethal essential for life l2efl |  | 3.7 | |
| DETOX_15_PI422610884 | ABCB4B | 2.0 | abc transporter |  | 2.9 | |
| CUST_8129_PI422575199 | AGAP000877-RA | 1.9 | cytochrome p450 |  | 2.2 | |
| DETOX_759_PI422610884 | SOD1 | 1.9 | superoxide mn |  | 4.3 | |
| DETOX_13_PI422610884 | ABCB4B | 1.7 | abc transporter |  | 2.7 | |
| DETOX_16_PI422610884 | ABCB4C | 1.7 | abc transporter |  | 2.6 | |
| DETOX_18_PI422610884 | ABCB4C | 1.6 | abc transporter |  | 2.5 | |
| DETOX_343_PI422610884 | CYP4C36 | 1.5 | cytochrome p450 |  | 2.7 | |
| **Top most down-regulated detoxification genes in mated not differentially expressed in resistant mosquitoes** | | | | | | |
| CUST_8352_PI422575199 | AGAP001039-RB | 2.9 | cytochrome p450 |  | |  |
| DETOX_256_PI422610884 | CYP307A1 | 2.6 | cytochrome p450 |  | |  |
| DETOX_178_PI422610884 | COEBE2O | 2.5 | juvenile hormone esterase |  | |  |
| DETOX_688_PI422610884 | NIT8537 | 2.4 | nitrilase member 2 |  | |  |
| DETOX_283_PI422610884 | CYP325C2 | 2.0 | cytochrome p450 |  | |  |
| CUST_11315_PI422575199 | AGAP008020-RA | 2.0 | cytochrome p450 |  | |  |
| DETOX_25_PI422610884 | ABCC12 | 2.0 | multidrug resistance-associated protein |  | |  |
| CUST_1241_PI422575199 | AGAP005774-RA | 1.9 | cytochrome p450 |  | |  |
| DETOX_214_PI422610884 | CYP12F1 | 1.9 | cytochrome p450 |  | |  |
| DETOX_338_PI422610884 | CYP4C28 | 1.9 | cytochrome p450 |  | |  |
| CUST_6435_PI422575199 | AGAP013121-RB | 1.8 | glucosyl glucuronosyl transferases |  | |  |
| DETOX_320_PI422610884 | CYP49A1 | 1.8 | cytochrome p450 |  | |  |
| DETOX_427_PI422610884 | CYP6AH1 | 1.7 | cytochrome p450 |  | |  |
| CUST_1214_PI422575199 | AGAP005751-RA | 1.7 | glucosyl glucuronosyl transferases |  | |  |
| DETOX_280_PI422610884 | CYP325C1 | 1.6 | cytochrome p450 |  | |  |
| DETOX_819_PI422610884 | TRX2 | 1.6 | mitochondrial thioredoxin 2 |  | |  |
| CUST_13790_PI422575199 | AGAP012667-RA | 1.6 | cytochrome p450 |  | |  |
| DETOX_251_PI422610884 | CYP305A4 | 1.6 | cytochrome p450 |  | |  |
| CUST_10103_PI422575199 | AGAP011507-RA | 1.6 | carboxylesterase |  | |  |
| DETOX_243_PI422610884 | CYP304C1 | 1.6 | cytochrome p450 |  | |  |
| DETOX_321_PI422610884 | CYP49A1 | 1.6 | cytochrome p450 |  | |  |
| DETOX_286_PI422610884 | CYP325C3 | 1.6 | cytochrome p450 |  | |  |
| DETOX_836_PI422610884 | XD18014 | 1.5 | aldehyde oxidase |  | |  |

**Table S10**: Expression profile of detoxification (or resistance-associated) genes up-regulated in resistant mosquitoes between mated and uncoupled males from Vallée du Kou

| **Probes** | **P value** | **FC** | **Genes** | **Function** | **Mated** | **Uncoupled** |
| --- | --- | --- | --- | --- | --- | --- |
| DETOX_487_PI422610884 | 0.0031 | 20.5 | CYP6Z2 | cytochrome p450 | / | / |
| DETOX_66_PI422610884 | 0.0088 | 16.6 | AGAP006226-RA | aldehyde oxidase | / | / |
| CUST_2219_PI422575199 | 0.0161 | 12.9 | AGAP006710-RA | chymotrypsin 1 | / | / |
| CUST_13058_PI422575199 | 0.0034 | 10.4 | AGAP009828-RA | chymotrypsin 1 | / | / |
| DETOX_461_PI422610884 | 0.0076 | 10.0 | CYP6P3 | cytochrome p450 | / | / |
| CUST_2218_PI422575199 | 0.0150 | 9.2 | AGAP006709-RA | chymotrypsin 1 | / | / |
| CUST_2696_PI422575199 | 0.0005 | 8.5 | AGAP007161-RA | lethal essential for life l2efl |  | 2.0 |
| DETOX_490_PI422610884 | 0.0262 | 4.9 | CYP6Z3 | cytochrome p450 | / | / |
| DETOX_742_PI422610884 | 0.0277 | 3.6 | PX7 | oxidase peroxidase | / | / |
| DETOX_706_PI422610884 | 0.0026 | 3.5 | PX13A | oxidase peroxidase | / | / |
| CUST_4820_PI422575199 | 0.0477 | 3.2 | CYP6P5 | cytochrome p450 | 1.74 | / |
| DETOX_748_PI422610884 | 0.0485 | 2.9 | PX9 | oxidase peroxidase | 1.62 | / |
| DETOX_34_PI422610884 | 0.0048 | 2.7 | ACE2 | acetylcholinesterase | / | / |
| DETOX_150_PI422610884 | 0.0209 | 2.7 | COEAE3G | alpha-esterase | / | / |
| CUST_6991_PI422575199 | 0.0019 | 2.5 | AGAP004380-RA | glutathione transferase gst1-6 | / | / |
| CUST_3911_PI422575199 | 0.0143 | 2.4 | CYP325B1 | cytochrome p450 | 1.53 | / |
| DETOX_410_PI422610884 | 0.0029 | 2.4 | CYP6AA1 | cytochrome p450 | / | / |
| DETOX_446_PI422610884 | 0.0251 | 2.4 | CYP6M4 | cytochrome p450 | / | / |
| DETOX_391_PI422610884 | 0.0391 | 2.4 | CYP4H26 | cytochrome p450 | 1.57 | / |
| CUST_8035_PI422575199 | 0.0055 | 2.4 | CYP9K1 | cytochrome p450 | / | / |
| CUST_514_PI422575199 | 0.0046 | 2.1 | AGAP005124-RA | aldehyde dehydrogenase | / | 2.4 |
| DETOX_425_PI422610884 | 0.0073 | 2.3 | CYP6AG2 | cytochrome p450 | / | / |
| CUST_11496_PI422575199 | 0.0090 | 2.3 | CYP6M2 | cytochrome p450 | / | / |
| DETOX_272_PI422610884 | 0.0110 | 2.3 | CYP325A2 | cytochrome p450 | 1.52 | / |
| DETOX_221_PI422610884 | 0.0087 | 2.3 | CYP12F3 | cytochrome p450 | / | / |
| DETOX_145_PI422610884 | 0.0076 | 2.2 | COEAE3D | carboxylesterase | 1.51 | / |
| DETOX_725_PI422610884 | 0.0220 | 2.2 | PX3 | oxidase peroxidase | 1.55 | / |
| DETOX_732_PI422610884 | 0.0407 | 2.2 | PX4B | oxidase peroxidase | 1.59 | / |
| CUST_4860_PI422575199 | 0.0107 | 2.2 | CYP6P2 | cytochrome p450 | / | 1.73 |
| DETOX_330_PI422610884 | 0.0428 | 2.2 | CYP4C25 | cytochrome p450 | 1.58 | / |
| DETOX_415_PI422610884 | 0.0337 | 2.1 | CYP6AD1 | cytochrome p450 | 1.78 | / |
| DETOX_299_PI422610884 | 0.0086 | 2.1 | CYP325F1 | cytochrome p450 | / | / |
| DETOX_607_PI422610884 | 0.0076 | 2.1 | GSTD8 | glutathione s-transferase | 1.51 | / |
| CUST_3883_PI422575199 | 0.0204 | 2.1 | AGAP002195-RA | cytochrome p450 | / | / |
| DETOX_268_PI422610884 | 0.0470 | 2.1 | CYP325A1 | cytochrome p450 | 1.56 | / |
| DETOX_301_PI422610884 | 0.0492 | 2.1 | CYP325F2 | cytochrome p450 | 1.54 | / |
| CUST_1736_PI422575199 | 0.0116 | 2.0 | AGAP005638-RA | aldehyde oxidase | / | / |
| DETOX_133_PI422610884 | 0.0156 | 2.0 | COEAE2A | carboxylesterase | 1.50 | / |
| CUST_9643_PI422575199 | 0.0410 | 2.0 | AGAP011028-RA | cytochrome p450 | / | / |
| DETOX_115_PI422610884 | 0.0098 | 2.0 | COE18026 | carboxylesterase | 1.57 | / |
| CUST_1097_PI422575199 | 0.0349 | 2.0 | AGAP005638-RA | aldehyde oxidase | 1.51 | / |
| DETOX_442_PI422610884 | 0.0417 | 2.0 | CYP6M3 | cytochrome p450 | / | / |
| DETOX_455_PI422610884 | 0.0096 | 2.0 | CYP6P1 | cytochrome p450 | / | / |

/ no differential expression
